# Clinical Application of Traditional Chinese Medicine Therapy for Type 2 Diabetes Mellitus: An Evidence Map

**DOI:** 10.1155/2022/2755332

**Published:** 2022-07-19

**Authors:** Ying Wang, Zelei Dai, Qin Wang, Ying He, Yalan Peng, Miaomiao Wu, Haiqi Song, Long Ma, Yonggang Zhang, Nian Li

**Affiliations:** ^1^Department of Medical Administration, West China Hospital, Sichuan University, Chengdu 610000, China; ^2^Department of Head and Neck Oncology, Cancer Center, West China Clinical School of Medicine, Sichuan University, Chengdu 610041, China; ^3^Department of Evidence-Based Medicine and Clinical Epidemiology, West China Hospital, Sichuan University, Chengdu 610041, China; ^4^Department of Integrated Traditional and Western Medicine, West China Hospital, Sichuan University, Chengdu 610041, China; ^5^Hospital Infection Control Department, West China Hospital, Sichuan University, Chengdu 610041, China; ^6^Department of General Practice and National Clinical Research Center for Geriatrics, International Medical Center, West China Hospital, Sichuan University, Chengdu 610041, China; ^7^The First Clinical Medical College of Gansu University of Chinese Medicine, Lanzhou 730000, China; ^8^Department of Periodical Press, West China Hospital, Sichuan University, Chengdu 610041, China; ^9^Chinese Evidence-Based Medicine Center, West China Hospital, Sichuan University, Chengdu 610041, China

## Abstract

Type 2 diabetes mellitus (T2DM), a common disease with a complex etiology in the world, is an important risk factor for severe cardiovascular and cerebrovascular diseases. However, treatments of T2DM are mainly based on Western medicine, whose severe side effects make traditional Chinese medicine (TCM) therapy more appealing to patients and clinicians. The overall clinical evidence for different TCM therapies in the treatment of T2DM is still unclear. This study aimed to adopt the evidence-mapping method and integrate the evidence from various researches on this topic, to depict the whole picture of TCM therapies for T2DM. This review included searches of PubMed, Embase, Web of Science, and three major Chinese literature databases (CNKI, VIP, and Wanfang) from inception to November 18, 2021. Two independent reviewers screened the literature, extracted information, and evaluated the quality of all included studies. A systematic review was subsequently performed. In total, 47 studies were reviewed, of which 46 studies (97.9%) were from China and 1 (2.1%) was from Canada. The evidence map was conducted according to different TCM therapies, including herbs or herbal extracts, compounds, powders, decoctions, pills, external treatment, basic theories and treatment principles of TCM, proprietary Chinese medicines, and unspecified TCM integrated therapies. According to the AMSTAR-2 scoring results, 4 papers were rated as high quality, 11 were low quality, and 32 were very low quality. Outcome indicators mainly focused on FBG, HbA1c, 2-h PBG, TC, TG, LDL-C, etc. The results showed that different types of TCM treatment had different improvement effects on the outcome indicators of T2DM. More consistent benefits were observed in the improvement of FBG, HbA1c, and 2-h PBG with treatment regimens based on basic theories and treatment principles of TCM, decoctions and pills, and unspecified TCM integrated therapies. Among herbs, ginger and Coptis root showed more improvement in all outcomes. Compounds, powders, and external treatment showed relatively consistent beneficial effects on the improvement of FBG. No serious adverse events were reported. Overall, the current evidence map provided an intuitive overview of the beneficial effects of TCM therapies in the treatment of type 2 diabetes. This study can be used as a reference for the clinical application of traditional Chinese medicine in T2DM, but due to the low-quality level of the included studies, it should be treated with caution in clinical practices.

## 1. Introduction

Type 2 diabetes mellitus (T2DM) is a common disease with a complex etiology around the world. The current development of diabetes has far exceeded expectations. The World Health Organization (WHO) reported that approximately 300 million people will suffer from diabetes in 2025 [1–3]. The trend of diabetes prevalence in China is the same as in the rest of the world. The prevalence of diabetes in Chinese adults as defined by WHO criteria increased from 9.7% in 2007 to 11.2% in 2017. In the April of 2020, a study showed that the prevalence of diabetes in Chinese adults was 12.8% and the total number of diabetic patients was about 129.8 million [4].

Diabetes is also an important risk factor for severe cardiovascular and cerebrovascular diseases, and T2DM can cause a variety of complications if blood glucose levels were not controlled, resulting in a serious decline in quality of life and high outpatient and inpatient costs. Real-time monitoring and effective control are required to reduce its burden.

Treatments for T2DM are mainly via Western medicine (antidiabetic drugs such as insulin and metformin) and lifestyle modification. However, severe side effects from medications make TCM complementary and alternative therapies more attractive to patients and clinicians [1, 5]. These TCM treatments include proprietary Chinese medicine, decoctions, pills, powders, herbal medicine, external treatment, and others [5].

A large number of systematic reviews and meta-analyses have been published on the TCM treatment for T2DM, and researchers often conducted clinical trials on a certain type of Chinese herbal medicine, proprietary Chinese medicine, and Chinese medicine compounds to verify its clinical effectiveness. However, the overall clinical evidence for different TCM classifications in the treatment of T2DM remains unclear.

Therefore, this study aims to reevaluate systematic reviews and meta-analyses, adopt the evidence mapping method, integrate the evidence from various research, and comprehensively sort out the problems in the research topic, thus depicting the whole picture of the research field [6]. No evidence map of T2DM has been published in the field of TCM. Therefore, this study used an evidence map to systematically retrieve the relevant literature (SRs) on the clinical treatment of T2DM, in order to better understand the distribution of evidence in this field and provide readers with more valuable and integrative evidence.

## 2. Materials and Methods

### 2.1. Search Strategy

Publication search was conducted in PubMed, Embase, Web of Science, and 3 major Chinese literature databases, including CNKI, VIP, and Wanfang Data (Supplementary Table 1 to Supplementary Table 6). A manual search of the unpublished literature (including conference proceedings, theses and dissertations, and gray literature) was also performed. The search time frame was from the inception to November 18, 2021, and the language was restricted to Chinese and English.

### 2.2. Inclusion Criteria

Inclusion criteria were as follows:

1. All meta-analyses and/or systematic reviews (SRs) on TCM treatments for T2DM were included.
2. Types of studies included in the SRs and meta-analyses should be RCTs.
3. Participants were diagnosed with T2DM, and there were no restrictions on age, gender, complications, or previous treatment.
4. The type of intervention was the use of at least one TCM therapy. There were no limitations on dosage, duration, and combined therapy.
5. The control group could be standard Western medicine treatment, placebo, or no treatment.
6. Primary outcomes included HbA1c, 2-h PBG, and FBG; secondary outcomes included BMI, HDL-C, HOMA-IR, LDL-C, TC, TG, INS, 2 h postprandial insulin, time to target blood glucose, average insulin dose, ISI, HOMA-*β*, hypoglycemia occurrence frequency/rate, clinical efficacy, TCM syndrome, etc.
7. No limitations were imposed on the study design or publication type.

### 2.3. Exclusion Criteria

(1) Clinical experts' experience, (2) clinical trial protocols, (3) meeting abstracts, (4) unavailable full text, (5) redundant publication, (6) fundamental research or pharmacological research of Chinese herbal medicine, and (7) animal studies were excluded.

### 2.4. Study Selection and Data Extraction

The authors (WY and DZL) screened the titles and abstracts of all retrieved references after removing duplicates, and the full text was obtained for further screening. WY checked all the eligible studies, and any disagreements were solved by a discussion with DZL, ZYG, and LN. The data extraction was conducted by WY and DZL and then validated by ZYG. The extracted information recorded was the title, authors, publication year, name of TCM, types of research included, the number of research included, interventions, controls, outcomes, effect value, etc. Disagreements were resolved by discussion, and a consensus was reached through a third party.

### 2.5. Quality Assessment

Two independent reviewers evaluated the quality of included studies. A measurement tool to assess SRs (AMSTAR-2) [7], which consists of 16 items, was used to evaluate the methodological quality of all the included SRs. For each item, when the evaluation criteria were completely satisfied, the result was “yes.” When the criteria were partially met, the evaluation result was “partially yes.” When no relevant information was reported in the SRs, the result was “no.” The key entries were 2, 4, 7, 9, 11, 13, and 15 [8]. If no or only one noncritical item failed, the quality level was high. If more than one noncritical item was not met, the quality level was medium. If one key item was not met, the quality rating was low. If more than one key item was not met, the quality level was very low.

### 2.6. Data Synthesis and Presentation

The quantitative description was conducted in Microsoft Excel 365. Data summary and analysis were shown as text and charts, distribution of the development trend was depicted as a line chart [9], and the distribution of evidence as bubble plots was conducted in python3 (matplotlib, pandas) [10].

## 3. Results

### 3.1. Literature Screening Process and Results

**Figure 1:**
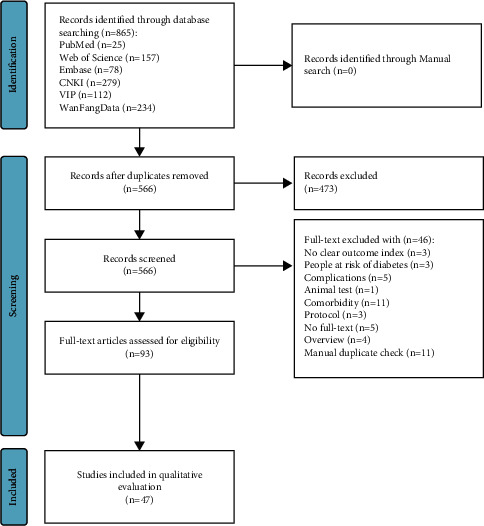
Literature screening process and results.

An initial review of 865 relevant sources was conducted. After removing duplicates, 566 studies were identified. After screening the titles and abstracts, 93 studies were retained. After screening the articles in full text, we further excluded 46 records, so a total of 47 studies [11–57] were reviewed (Figure 1).

### 3.2. The Basic Information of the Included Literature

The basic information of the included literature is listed in Table 1; 46 studies (97.9%) were from China [11–33, 35–57], and 1 (2.1%) was from Canada [34]. According to different TCM therapies, they were divided into herbs or herbal extracts, compounds, powders, decoctions, pills, external treatment, basic theories and treatment principles of TCM, proprietary Chinese medicines, and unspecified TCM integrated therapies (Table 2). According to the AMSTAR-2 scoring results, 4 papers were assessed as high quality, 11 were low quality, and 32 were very low quality (Supplementary Table 7).

### 3.3. Bibliometric Information and Characteristics of the Included RCTs

**Figure 2:**
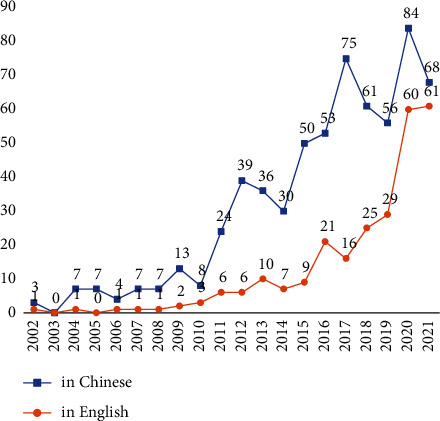
Number of the systematic review publications on TCM treatment for T2DM.

In Figure 2, the increasing overall trend in the number of studies was demonstrated. Before 2010, reviews were conducted sporadically. The number increased rapidly after 2010, with up to 144 systematic reviews published in one year.

Table 3 shows the outcome indicators of TCM treatments of T2DM, the number of systematic reviews corresponding to the different indicators, and the number of comparative analyses with other control measures.

### 3.4. Effects of the TCM Therapies on T2DM

**Figure 3:**
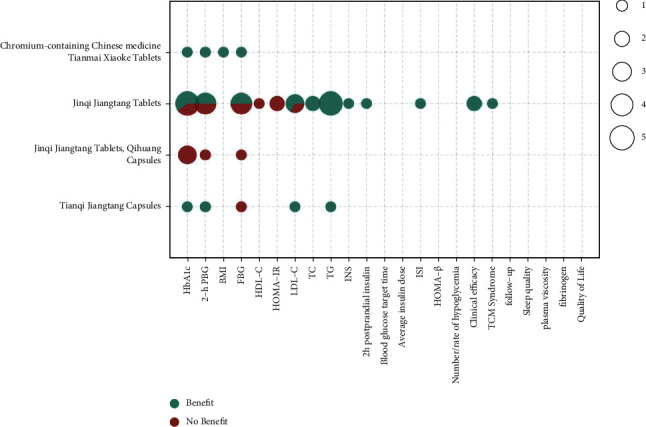
Outcomes and effects of proprietary Chinese medicines (horizontal axis: T2DM outcome indicators; ordinate: TCM; dark green color: beneficial effect; dark red color: nonbeneficial effect; bubble area: number of meta-analysis for intervention-control comparisons).

**Figure 4:**
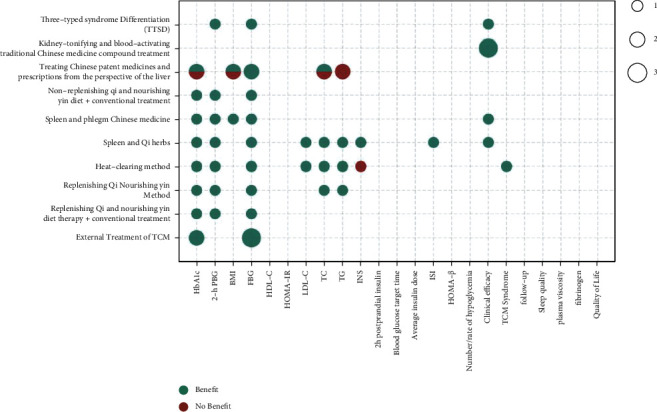
Outcomes and effects of basic theories and treatment principles of TCM.

**Figure 5:**
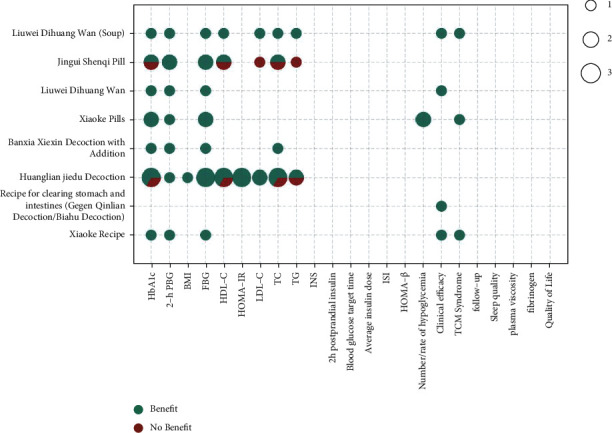
Outcomes and effects of decoctions and pills.

**Figure 6:**
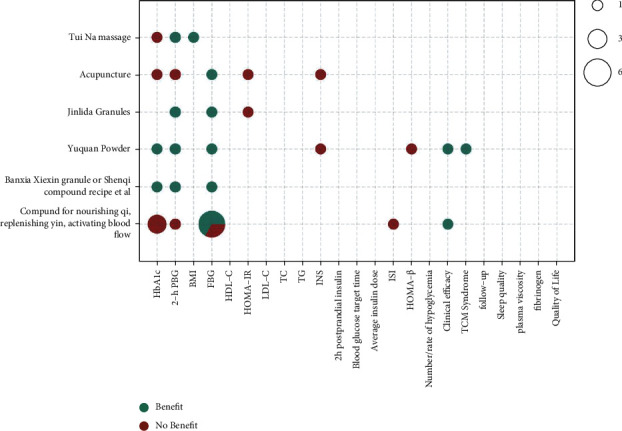
Outcomes and effects of compounds, powder, and external treatment methods (Banxia Xiexin granules, compound of Ginseng-Astragalus, tonifying Qi and strengthening spleen decoction, and Gegen Qinlian decoction.).

**Figure 7:**
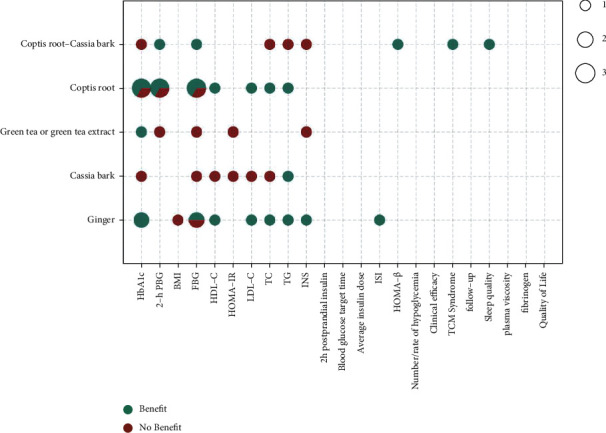
Outcomes and effects of herbal medicines.

**Figure 8:**
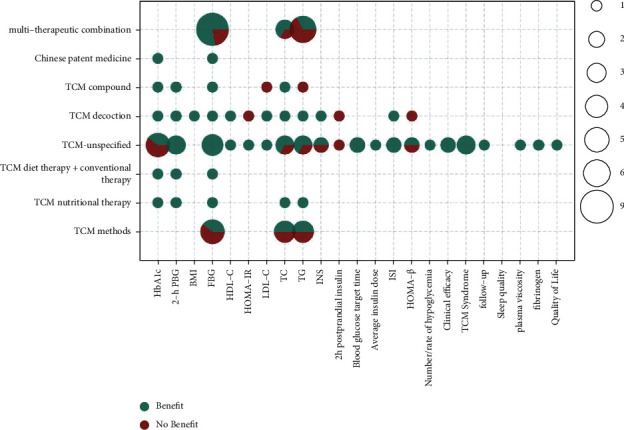
Outcomes and effects of integrated TCM therapies.

The metrics of clinical outcomes and intervention are shown in Figures 3–8 according to different TCM classifications. The consistent beneficial effect according to the systematic reviews and meta-analysis are shown in dark green. Nonmeaningful outcomes are shown in dark red, and the numbers of intervention-control comparisons for each outcome are shown as the bubble area.

### 3.5. Proprietary Chinese Medicines

Of the 47 included systematic reviews, a total of 6 papers [21, 22, 37, 42, 51, 57] compared the effects of TCMs such as Tianqi Jiangtang capsules, Jinqi Jiangtang tablets, Jinqi Jiangtang tablets and Qihuang capsules, and chromium-containing Chinese herbal medicine Tianmai Xiaoke tablets on the treatment of T2DM by meta-analysis. Improvements in various outcome indicators did not show a more consistent beneficial effect than conventional or placebo therapy alone. Improvements in glycosylated hemoglobin (5/10), 2-hour postprandial glucose (4/7), and FBG (3/7) showed beneficial effects in only half of the studies, with consistent beneficial effects only on TG (6/6).

### 3.6. Basic Theories and Treatment Principles of TCM

A total of 10 [14, 17, 19, 20, 24, 28, 40, 48, 55, 56] of the 47 included systematic reviews compared the effectiveness of basic theories and treatment principles of TCM, including external treatment of TCM, nourishing Qi, replenishing yin, activating blood flow, replenishing Qi and nourishing yin diet therapy + conventional treatment, replenishing Qi nourishing yin method, heat-clearing method, tonifying spleen, and Qi herbs, strengthening the spleen, and reducing phlegm Chinese medicine, nonreplenishing Qi, and nourishing yin diet therapy + conventional treatment, treating proprietary Chinese medicines and prescriptions from the perspective of the liver, kidney-tonifying and blood-activating TCM compound treatment, and TTSD on the treatment of T2DM by meta-analysis. Improvements in all outcome indicators relative to conventional therapy or placebo therapy alone showed consistent beneficial effects. Improvements in glycated hemoglobin (9/10), 2-hour postprandial glucose (7/7), FBG (12/12), and clinical outcomes (6/6) were all highly beneficial. There were also beneficial effects on the improvements of BMI, LDL-C, TC, INS, ISI, and TCM syndrome.

### 3.7. Decoctions and Pills

A total of 11 [11, 15, 16, 23, 25, 27, 29, 38, 45, 47, 53] of the 47 included systematic reviews compared the effects of Xiaoke recipe, the recipe for clearing stomach and intestines (Gegen Qinlian decoction/Baihu decoction), Huanglian Jiedu decoction, Banxia Xiexin decoction with addition, Xiaoke pills, Liuwei Dihuang Wan, Jingui Shenqi pill, and Liuwei Dihuang Wan (soup) on the treatment of T2DM by meta-analysis. Improvements in all outcome indicators relative to conventional therapy or placebo therapy alone showed consistent beneficial effects. Improvements in glycated hemoglobin (9/11), 2-hour postprandial glucose (8/8), FBG (11/11), HOMA-IR (3/3), clinical outcomes (4/4), and TCM symptoms (3/3) were all highly beneficial. It was also beneficial for the improvements of BMI, HDL-C, LDL-C, TC, TG, and incidence of hypoglycemia.

### 3.8. Compounds, Powder, and External Treatment Methods

Of the 47 included systematic reviews, a total of 6 papers [24, 36, 44–46] compared the efficacy of the compound of nourishing Qi, replenishing yin, and activating blood flow, a compound of Ginseng-Astragalus, Yuquan powder, Jinlida granules, Banxia Xiexin granules, acupuncture, and Tui Na on the treatment of T2DM by meta-analysis. The overall beneficial effect on improvements of all outcome indicators was not significant relative to conventional or placebo therapy alone. There was a consistent improvement effect on FBG (8/10), with some beneficial effects on BMI, clinical outcomes, and TCM syndrome. However, there were inconsistent and insignificant improvement effects on glycated hemoglobin (2/7), 2-hour postprandial glucose (4/6), HOMA-IR (0/2), INS (0/2), ISI (0/1), and HOMA- *β* (0/1).

### 3.9. Herbal Medicine

A total of 6 [13, 26, 34, 35, 43, 49] of the 47 included systematic reviews compared the effects of ginger, cassia bark, green tea or green tea extract, Coptis root, and Coptis-cassia bark by meta-analysis for the treatment of T2DM. Coptis root showed beneficial effects on glycated hemoglobin, 2-hour postprandial glucose, FBG, HDL-C, TC, and TG in 10 of 13 meta-analyses. Ginger showed beneficial effects on glycated hemoglobin, FBG, HDL-C, LDL-C, TC, TG, INS, and ISI in 9 of 11 meta-analyses.

### 3.10. Integrated TCM Therapies

A total of 11 [58, 76–79, 85, 87, 96, 98, 100, 102] of the 47 included systematic evaluations compared the effects of unspecified TCM, including TCM methods such as Baduanjin, TCM nutritional therapy, TCM diet and conventional therapy, other unspecified TCM, TCM decoction, TCM compound, Chinese patent medicine, and multitherapeutic combination on T2DM by meta-analysis. Relative to conventional therapies alone, the integrated TCM therapies showed a more consistent beneficial effect on the improvement of glycosylated hemoglobin (7/10), with only 3 unspecified TCMs being insignificant in the 10 comparative studies. They showed a completely consistent beneficial effect on the improvement of 2-hour postprandial glucose (7/7) and showed a more consistent beneficial effect on the improvement of fasting glucose (18/23), with 2 insignificant multitherapeutic combinations and 3 insignificant TCM methods. The improvement in TC showed a more consistent beneficial effect (9/13); 2 TCM methods were not significant, 1 TCM were unspecified, and 1 multitherapeutic combination was not significant. For the improvement in TG, only half of the studies showed a beneficial effect (8/16), and for improvement in 2 hours' postprandial insulin, the integrated TCM therapies did not show a beneficial effect. The effects of TCM compound, Chinese patent medicine, and TCM unspecified on improving BMI, HDL-C, HOMA-IR, LDL-C, INS, blood glucose target time, average insulin dose, ISI, HOMA-*β*, number/rate of hypoglycemia, and clinical efficacy, TCM syndrome, follow-up, sleep quality, plasma viscosity, fibrinogen, and quality of life all had beneficial effects.

### 3.11. Adverse Events

In the intervention group using TCM therapies, there were few reports of adverse reactions, most of which were incidental cases, and no serious adverse reactions were reported. Zhipeng Hu [11] remarked that one RCT reported one case of headache, three cases of nausea and vomiting, one case of dizziness, and one case of dry cough in the TCM treatment group and one RCT reported one case of hypoglycemia and four cases of mild nausea and loss of appetite, which then gradually disappeared. Jiarong Lan [13] mentioned that no serious adverse reactions affecting vital organs occurred during the experiment. The incidence of adverse reactions was dose-dependent. Chi Xiao [18] noted that one RCT reported one case of the hypoglycemic reaction occurred in the TCM treatment group after 2 weeks of treatment, while 2 cases occurred in the control group. Some RCT studies reported possible GI adverse reactions in both the TCM treatment and control groups [21]. One RCT reported a total of 3 cases of gastrointestinal (GI) adverse reactions, but none of them required special treatment [22]. It has been summarized [24] that some RCTs reported the occurrence of adverse reactions, such as mild nausea and vomiting, mild gastrointestinal discomfort, mild hypoglycemia, skin pruritus, and mild tingling in the ear in a few patients. Jiahui Hu [39, 40], Huijuan Gao [42], Aiping Deng [50], and Jiaxing Tian [52] noted that there was no statistically significant difference in the incidence of adverse reactions between the TCM treatment group and the control group. Ying Fu [41] mentioned that the studies involved all GI reactions. Another review concluded [47] that hypoglycemic events were reported in the RCT studies, but there was no significant difference between the control group (6 cases) and the TCM treatment group (5 cases). Jiang Li [48] alluded that one RCT reported 6 cases of hyperthermia in the control group and 4 cases of vomiting in the TCM group, and another RCT reported one case of hypoglycemia in both groups. One literature reported the incidence of GI adverse reactions in the TCM group was 2.5% while that in the control group was 7.5% [48]. It has been indicated [53] that the combination of Liu Wei Di Huang Wan (soup) with metformin for T2DM has the potential to reduce the adverse effects of metformin. Yuming Gu [57] mentioned that the common adverse events in the TCM group were gastrointestinal symptoms (nausea/vomiting, bloating, and diarrhea), neurological symptoms, and hypoglycemia. However, no significant abnormalities in blood, liver, or kidney functions were seen in all studies.

## 4. Discussion

### 4.1. Summary of Findings

The evidence map for TCM treatments of T2DM was developed based on 47 SRs and meta-analyses, providing an evidence overview of the impact of different TCM therapies on different outcomes. The included literature was for patients with only T2DM but not comorbidities and complications. There were many systematic reviews on Coptis root, Jinqi Jiangtang tablets, Liuwei Dihuang pills, Xiaoke pills, and methods of nourishing Qi, replenishing yin, and activating blood flow. The outcome indicators mainly focus on FBG, HbA1c, 2-h PBG, TC, TG, and LDL-C. The results showed that different types of TCM treatments had different improvement effects on the outcome indicators of T2DM. More consistent benefits were observed in the improvement of the primary outcomes with basic theories and treatment principles of TCM, decoctions and pills, and unspecified TCM integrated therapies. Among the herbal medicine, ginger and Coptis root showed benefits in all outcomes. The improvement of FBG was more consistent with compounds, powders, and external treatment. No report of serious adverse reactions has been found, but mild gastrointestinal reactions were common, such as nausea, vomiting, abdominal distension, and diarrhea, and they did not require special treatments.

### 4.2. Differences from Previous Studies

The method of using the evidence map to provide evidence summary has been used in the treatment of hypertension with TCM [55], and there have been reviews published in English on the treatment of diabetes with TCM [1]. This paper is the first evidence map systematic review on the treatment of T2DM with TCM.

### 4.3. Limitations

The study has some limitations. First, most systematic reviews of TCM research were published in Chinese, which may have publication bias. Second, in all SRs and meta-analyses included, only a few studies were of high quality, and most were of low or very low quality.

### 4.4. Implications for Future Research

The study provides enlightenment for the future TCM treatments of diabetes and related academic research. First, the results of FBG showed the most consistent beneficial effect among different TCM treatments, which was beneficial for long-term control and prevention of complications. Second, in most of the included SRs and meta-analyses, TCM therapies were used in combination with conventional Western medicine treatment, but the different combinations of TCM and Western medicine may achieve different results and further clinical studies are needed. Third, a few studies found that TCM may reduce the side-effects of Western medicine, but the evidence was not clear enough. The interaction between TCM and Western medicine needs to be explored.

### 4.5. Implications for Clinical Practice

In China, emphasizing the application of both TCM and Western medicine has become a national strategy. But in clinical practice, the indications of TCM therapies are currently too broad, and the current evidence cannot convince clinicians to choose a specific treatment method. The evidence map shows that the external treatment of TCM, replenishing Qi and nourishing yin diet therapy + conventional treatment, replenishing Qi nourishing yin method, heat-clearing method, tonifying spleen and Qi herbs, strengthening the spleen and reducing phlegm Chinese medicine, nonreplenishing Qi and nourishing yin diet therapy + conventional treatment, TTSD, Xiaoke recipe, Banxia Xiexin decoction, Xiaoke pills, Liuwei Dihuang Wan (soup), and chromium-containing Chinese herbal medicine Tianmai Xiaoke tablets have a relatively clear application value for hypoglycemia and can be used as a careful reference for making diagnosis and treatment plans in clinical practice. However, the combined usage, administration time, and duration of TCM therapies still need to be further explored. Given the promising findings in the evidence map from this paper, the use of these TCM therapies may become more widespread if supported by further well-designed trials or real-world studies.

## 5. Conclusions

Overall, the evidence map provides an intuitive overview of the beneficial effects of TCM therapies in the treatment of T2DM. This study can be used as a reference for the clinical application of TCM in T2DM, but due to the relatively low-quality level of the included studies, it is recommended to be used with caution in clinical practice. This study also enlightens the future research direction of evidence-based medicine and further study about the clinical application of TCM.

## Figures and Tables

**Table 1:** The basic information of TCM treatment for T2DM.

Author	Country	Research department	TCM treatment	Contains ingredients/contents	Number of RCTs included
Zhipeng Hu 2021 [11]	China	Affiliated Hospital of Chengdu University of Chinese Medicine	Huanglian Jiedu decoction	Phellodendron chinense Schneid. Rutaceae (Huáng B˘ai) and Gardenia jasminoides Ellis Rubiaceae (Zhi Zĭ).	9
Dingyuan Zhong 2016 [12]	China	Guangzhou University of Chinese Medicine	Unspecified TCM therapy	—	13
Jiarong Lan 2015 [13]	China	Wenzhou Hospital of TCM, Zhejiang University of TCM	Coptis root	—	27
Xue Wang 2015 [14]	China	Shandong University of TCM	Tonifying spleen and Qi herbs	Buzhong Yiqi decoction Flavored, Buzhong Yiqi decoction modified, spleen-invigorating, hypoglycemic decoction, spleen-invigorating, and Qi-reducing decoction, spleen-invigorating and Qi-reducing Chinese medicine, spleen-reducing sugar decoction, spleen-invigorating, and Qi-reducing TCM, self-made health spleen hypoglycemic formula, Jianpi Jiangtang decoction, “pancrease kangxiao” capsules, Chinese herbs for strengthening spleen and Qi	11
Haibo Huang 2015 [15]	China	Hunan University of TCM	Liuwei Dihuang Wan	—	8
Yongzhong Wang 2015 [16]	China	The First Affiliated Hospital of Anhui University of TCM	Xiaoke pills	—	15
Xiaodong Han 2014 [17]	China	Hebei Medical University	Compound of nourishing Qi, replenishing Yin, and activating blood flow	—	19
Chi Xiao 2014 [18]	China	Dalian Medical University	Yuquan powder	—	10
Xiu-Feng Yan 2014 [19]	China	Guang'anmen Hospital	Three-typed syndrome differentiation (TTSD)	—	19
Lu sun 2012 [20]	China	Guangdong Provincial Hospital of TCM	Kidney-tonifying and blood-activating TCM compound treatment	—	15
Winnie Chen 2012 [21]	China	Guangdong University of Chinese Medicine	Jinqi Jiangtang tablets, Qihuang capsules	—	17
Jinlan Peng 2013 [22]	China	Department of Endocrinology, Pu'ai Hospital, Tongji Medical College, Huazhong University of Science and Technology	Jinqi Jiangtang tablets	*Astragalus*, Huanglian, and honeysuckle	6
Pu Run 2013 [23]	China	Department of Pharmacy Management and Clinical Pharmacy, School of Pharmacy, Peking University Medical Center	Liuwei Dihuang Wan	—	16
Luyao Zhang 2019 [24]	China	Beijing University of Chinese Medicine	Compound of nourishing Qi, replenishing Yin, activating blood flow	—	17
			External treatment of TCM : nourishing Qi, replenishing Yin, activating blood flow	—	5
Yongzhong Wang 2014 [25]	China	Anhui University of TCM, First Affiliated hospital	Xiaoke pills	—	17
Jie Zhu 2018 [26]	China	The Second Clinical College, Nanjing University of Chinese Medicine, Nanjing, Jiangsu 210023, China	Ginger	—	Qualitative 12, meta 10
Jingyan Yan 2018 [27]	China	Jiangxi University of TCM Research Center for the Development of Differentiated Basic Theories of TCM	Recipe for clearing stomach and intestines (Gegen Qinlian Decoction/Bai-hu decoction)	—	24
Yuqing Liang 2018 [28]	China	School of TCM, Jinan University	Strengthen spleen and reduce phlegm Chinese medicine	Shenling Baizhu powder modified, Wuling powder modified, TCM decoction for strengthening spleen and dampness, modification and subtraction of Jianpi Yiqi Huazhuo Fang, Heqi powder, Xiaoke Jianpi capsule, Wuling powder modification, and subtraction, Shenling Baizhu powder modified, modified Wuling powder, modified Shenling Baizhu powder, invigorating the spleen and removing dampness	12
Wei Zhang 2018 [29]	China	Yinchuan Hospital of TCM	Xiaoke recipe	—	18
Yue Cheng 2018 [30]	China	T2DM	TCM compound	—	14
Chongqi Ma 2017 [31]	China	North China University of Technology	TCM nutritional therapy	—	11
Linlin Kong 2012 [32]	China	School of Business Administration, Shenyang Pharmaceutical University	TCM methods	Compound pancreatic Suling, Liu Wei di Huang Wan, Xian Hu capsules, low phenolic cotton seed capsules, anti-thirst spirit, Tui Na Chiropractic	7
			Multitherapeutic combination		12
Tingting Shao 2009 [33]	China	Chengdu University of Chinese Medicine	Unspecified TCM therapy		12
Dugoua Jean-Jacques 2007 [34]	Canada		Cassia bark	cassia bark	3
X. Wang 2014 [35]	China	Key Laboratory of Endocrinology, Ministry of Health, Department of Endocrinology, Peking Union Medical College Hospital, Peking Union Medical College, Chinese Academy of Medical Sciences	Green tea or green tea extract	Green tea or green tea extract	7
Xiaolin Zhang 2020 [36]	China	College of Acupuncture and Massage, Changchun University of Chinese Medicine	Tui Na (massage therapy)	Chinese massage (CM)	10
Chunli Piao 2020 [37]	China	Institution of Shenzhen Hospital, Guangzhou University of Chinese medicine	Tianqi Jiangtang capsules	Tianqi hypoglycemic capsules	8
Xitao Ma [38]	China	Chengdu University of Traditional Chinese Medicine	Banxia Xiexin decoction with addition	—	9
Jiahui Hu 2020 [39]	China	Beijing University of Chinese Medicine	Chinese patent medicine	Liu Wei di Huang Wan, Xiao Xie Wan, Jin Li da, Ginseng-Astragalus hypoglycemic granules/capsules/tablets (abbreviated as Ginseng-Astragalus hypoglycemic), Tian Qi hypoglycemic capsules/granules/tablets	42
Jiahui Hu 2020 [40]	China	Beijing University of Chinese Medicine	Treating proprietary Chinese medicines and prescriptions from the perspective of the liver	Prosperity san\diversion of the liver and qi herbs\nourishing Yin and draining the liver herbs\liver and spleen harmonizing decoction\detoxification and liver regulating formula\digestion and liver clearing drink\Dan Gardenia prosperity san\liver clearing and heart diarrhea decoction\added flavor da Chai Hu granules\sugar Min Ling Wan\liver diarrhea and dampness formula	13
Ying Fu 2020 [41]	China	Nanjing University of Chinese Medicine	Unspecified TCM therapy	Huanglian Su, clearing heat, benefiting Qi and invigorating blood, clearing liver and lungs, Jinmai warming gall bladder, Huangzen Tang, Dahuang Huanglian diarrhea heart, Ge Gen cenlian Tang, Warming Yang, benefiting Qi and invigorating blood, clearing heat, invigorating blood, and resolving phlegm, Fuhe liver, and benefiting spleen	13
Huijuan Gao 2019 [42]	China	Yuquan Hospital, Tsing Hua University,	Jinqi Jiangtang tablets	Jinqi Jiangtang tablet	17
Fangyan Huang 2019 [43]	China	Youjiang Medical University for Nationalities	Ginger	Zingiber officinale	8
Fengmei Lian 2019 [44]	China	Department of Endocrinology, Guang'anmen Hospital,	Jinlida granules	Jinlida granules	15
Yujiao Zheng 2021 [45]	China	Department of Endocrinology, Guang' anmen Hospital, China Academy of Chinese Medical Sciences, Beijing, China	Banxia Xiexin granules	(Pinelliae Rhizoma 12 g, Scutellariae Radix 9 g, Coptidis Rhizoma 3 g, Ginseng Radix et Rhizoma 9 g, Zingiberis Rhizoma 9 g, Glycyrrhizae Radix et Rhizoma 9 g, Jujubae fructus 9 g),	4
			Compound of Ginseng	Ginseng Radix et Rhizoma 10 g, Astragali Radix 30 g, Corni fructus Radix 10 g, Rehmanniae Radix 15 g, Salviae et Rhizoma 6 g)	
			Gegen Qinlian decoction	(Puerariae Lobatae Radix, Scutellariae Radix, Coptidis Rhizoma, Glycyrrhizae Radix et rhizoma),	
			Tonifying Qi and strengthening spleen decoction	AMC herbal formula (Anemarrhenae rhizoma, Momordica charantia, Coptidis rhizoma, Salviae miltiorrhizae Radix et Rhizoma, fermentum Schisandrae chinensis fructus, and Zingiberis Rhizoma)	
Xu Zhou 2021 [46]	China	Evidence-Based Medicine Research Center, Jiangxi University of TCM, Nanchang, 330004, China.	Acupuncture	Acupuncture	21
Zhipeng Hu 2021 [47]	China	Hospital of Chengdu University of TCM, Chengdu, China	Jingui Shenqi pills	Rehmannia glutinosa (Gaertn.) DC. (dì Huáng), Orobanchaceae; dioscorea oppositifolia L. (Huái Shān Yào), Dioscoreaceae; cornus officinalis siebold (Shān Zhū Yú), Cornaceae; alisma plantago-aquatica L. (Zé Xiè), Alismataceae; Smilax glabra roxb. (Fú Líng), Smilacaceae; Paeonia × suffruticosa Andrews. (Mŭ dān Pí), Paeoniaceae; Neolitsea cassia (L.) Kosterm. (Guì Zhī), Lauraceae; and Aconitum carmichaelii debeaux, Ranunculaceae.	14
Jiang Li 2021 [48]	China	Dongzhimen Hospital Affiliated to Beijing University of Chinese Medicine, Beijing, China.	Heat-clearing method	*Astragalus*, yam, poria, atractylodes, and Ginseng; Coptis, rhubarb, Radix Scutellariae, sophora flavescens, and honeysuckle	15
Guohua Mu 2021 [49]	China	Beijing University of Chinese Medicine	Coptis-cassia bark	Huanglian-cassia bark	6
Aiping Deng 2021 [50]	China	Heilongjiang University of TCM	TCM decoctions	Benefiting Qi and nourishing Yin, hypoglycemic and sensitizing decoction *∗* 2, dan gardenia and lipid regulating decoction, beneficial Yin and dampness Chinese herbs, strengthening the spleen and kidneys, clearing phlegm and blood, warming the Yang and strengthening the spleen method, strengthening the spleen and benefiting Qi Chinese herbs, Tonifying the organs and enlarging the ligaments, Erzhu Xia Lan Tang, beneficial Qi and nourishing Yin and clearing heat, dispelling pancreatic resistance, warming the Yang and strengthening the spleen decoction, Ling Gui Jie Gan Tang	14
Zhiyuan deng 2020 [51]	China	Guangzhou University of Chinese Medicine First Clinical School of Medicine	Jinqi Jiangtang tablets	Golden Astragalus hypoglycemic tablets	10
Jiaxing Tian 2019 [52]	China	Tian, Jiaxing Department of Endocrinology, Guang'anmen Hospital, China Academy of Chinese Medical Sciences, Beijing, China	Unspecified TCM therapy	Pueraria mirifica powder, argyle sugar health tablets, anti-thirst pill, Kai Yu Qing heat and lowering turbidity formula, Tian Qi lowering sugar capsules, Jin Li da, sugar min Ling pill, Wu Mei formula, Tang Ke soft capsules, Pueraria Mirifica Scutellaria Tang, Huang Lian Su	12
Siyi Zhao 2019 [53]	China	Siyi Zhao, School of Acupuncture and Rehabilitation Clinical Medicine, Guangzhou University of TCM	Liuwei Dihuang Wan (soup)	Cornus officinalis, Chinese Yam, radix Rehmanniae, Radix Zeligae, dampi, poria	20
Tingting Guo [54]	China	Department of Pharmacy, Affiliated Hospital of Changchun University of TCM	Unspecified TCM therapy	—	12
Huiping Tian 2019 [55]	China	Department of Pharmacy, The First Affiliated Hospital of Xi'an Jiaotong University	Replenishing Qi nourishing Yin method	Yiqi Yangyin recipe, Sanqi dan granules, Yiqi Yangyin clearing heat and blood activating TCM, Yiqi Yangyin Huoxue Tongluo decoction, Yiqi Yangyin decoction, Quyi Difang decoction, Yiqi Yangyin Huoxue decoction, sugar kidney 1 no. Fang, Yiqi Yangyin Qingre Huoxue Fang	13
Yanling Dai 2019 [56]	China	School of Nursing, Fujian University of TCM; People's Hospital of Fujian University of TCM	TCM diet therapy + conventional therapy	—	12
			Replenishing Qi and nourishing Yin diet therapy + conventional treatment		
			Nonreplenishing Qi and nourishing Yin diet therapy + conventional treatment		
Yuming Gu 2018 [57]	China	Shandong University of TCM	Chromium-containing Chinese medicine Tianmai Xiaoke tablets	Tianmai Xiaoke tablet contains chromium picolinate (1.6 mg per tablet, equal to 200 *μ*g of chromium), Tianhuafen (Radix Trichosanthis, snake gourd root), Maidong (radix ophiopogonis, Dwarf Lilyturf tuber), and Wuweizi (fructus Schisandrae Chinensis)	7

**Table 2:** TCM therapy classification.

Classification	Content
Herbs or herbal extracts	Ginger, cassia bark, green tea or green tea extract, Coptis root, Coptis-cassia bark
Compounds	Compound of nourishing Qi, replenishing Yin, activating blood flow compound of Ginseng-Astragalus
Powder	Yuquan powder, Jinlida Granules, Banxia Xiexin Granules
Decoction	Xiaoke recipe, recipe for clearing stomach and intestines (Gegen Qinlian decoction/Baihu decoction), Huanglian Jiedu decoction, Banxia Xiexin decoction with addition, Gegen Qinlian decoction, Tonifying Qi and strengthening spleen decoction
External treatment	Acupuncture, Tui Na (massage therapy)
Pills	Xiaoke pills, Liuwei Dihuang Wan, Jingui Shenqi pill
Pills or decoction	Liuwei Dihuang Wan (soup)
Basic theories and treatment principles of TCM	External treatment of TCM, replenishing Qi and nourishing Yin diet therapy + conventional treatment, replenishing Qi nourishing Yin method, heat-clearing method, tonifying spleen, and Qi herbs, strengthen spleen and reduce phlegm Chinese medicine, nonreplenishing Qi and nourishing Yin diet therapy + conventional treatment, treating proprietary Chinese medicines and prescriptions from the perspective of the liver, kidney-tonifying and blood-activating TCM compound treatment, three-typed syndrome differentiation (TTSD)
Proprietary Chinese medicines	Tianqi Jiangtang capsules, Jinqi Jiangtang tablets, Qihuang capsules, Jinqi Jiangtang tablets, chromium-containing Chinese medicine Tianmai Xiaoke tablets
Integrated TCM therapies	TCM methods, TCM nutritional therapy, TCM diet therapy + conventional therapy, unspecified TCM therapy, TCM decoction, TCM compound, Chinese patent medicine, multitherapeutic combination

**Table 3:** Number of studies and comparative analysis corresponding to different outcome indicators.

Outcomes	Number of studies	Number of meta-analyses compared to the control group
HbA1c	40	56
2-h PBG	33	40
BMI	7	8
FBG	42	71
HDL-C	9	12
HOMA-IR	9	11
LDL-C	14	16
TC	21	31
TG	21	35
INS	11	11
2 h postprandial insulin	3	3
Blood sugar target time	2	2
Average insulin dose	1	1
ISI	7	7
HOMA-*β*	5	5
Number/rate of hypoglycemia	3	3
Clinical efficacy	13	16
TCM syndrome	10	10
Follow-up	1	1
Sleep quality	1	1
Plasma viscosity	1	1
Fibrinogen	1	1
Quality of life	1	1

## Supplementary Materials

Supplementary Materials Supplementary Table 1: search strategy of CNKI (279). Supplementary Table 2: search strategy of VIP (112). Supplementary Table 3: search strategy of Wanfang Data (234). Supplementary Table 4: search strategy of PubMed (25). Supplementary Table 5: search strategy of Web of Science (157). Supplementary Table 6: search strategy of Embase (78). Supplementary Table 7: AMSTAR-2 scoring results.
