# Supplementary material for: Clinical Application of Traditional Chinese Medicine Therapy for Type 2 Diabetes Mellitus: An Evidence Map
Source: Evid Based Complement Alternat Med. 2022 Jul 19;2022:2755332. doi: 10.1155/2022/2755332 (PMC9325645; doi:10.1155/2022/2755332)
Supplement: Supplementary Materials — Supplementary Table 1: search strategy of CNKI (279). Supplementary Table 2: search strategy of VIP (112). Supplementary Table 3: search strategy of Wanfang Data (234). Supplementary Table 4: search strategy of PubMed (25). Supplementary Table 5: search strategy of Web of Science (157). Supplementary Table 6: search strategy of Embase (78). Supplementary Table 7: AMSTAR-2 scoring results. [file 2755332.f1.docx]

# Supplementary table 1: Search strategy of CNKI (279)

| STEPS | Search Terms (in Chinese) |
| --- | --- |
| 1 | ( ( ( ( ( ( topic='Chinese medicine" or keyword= ('Chinese medicine') or title= ('Chinese medicine') or abstract= ('Chinese medicine') ) OR ( topic='Chinese herbal medicine' or keyword= ('Chinese herbal medicine') or title= ('Chinese herbal medicine') or abstract= ('Chinese herbal medicine') ) ) OR ( subject='Chinese herbal medicine' or keyword= ('Chinese herbal medicine') or title= ('Chinese herbal medicine') or abstract= ( 'TCM') ) ) OR ( subject='TCM' or keyword= ('TCM') or title= ('TCM') or abstract= ('TCM') ) ) OR ( subject='TCM' or keyword= ('Proprietary Chinese Medicine') or title= ('Proprietary Chinese Medicine') or abstract= ('Proprietary Chinese Medicine') ) ) AND ( ( Subject='Diabetes' or keyword= ('Diabetes') or title= ('Diabetes') or abstract= ('diabetes') ) OR ( subject='diabetes' or keyword= ('diabetes') or title= ('diabetes') or abstract= ('diabetes') ) ) ) AND ( ( subject='treatment ' or keyword= ('Treatment') or title= ('Treatment') or abstract= ('Treatment') ) OR ( Subject='Treatment' or keyword= ('Treatment') or title= ('Treatment') or abstract= ('diagnosis and treatment') ) ) ) AND ( ( ( subject='systematic review' or keyword= ('systematic review') or title= ('systematic review') or abstract= ('systematic review') ) OR ( subject='meta-analysis' or keyword= ('meta-analysis') or title= ('meta-analysis') or abstract= ('meta-analysis') ) ) AND ( subject='meta-analysis' or keyword= ('meta-analysis ') or title= ('meta-analysis') or abstract= ('meta-analysis') ) ) ) |

# Supplementary table 2: Search strategy of VIP (112)

| STEPS | Search Terms (in Chinese) |
| --- | --- |
| 1 | ((((((((((((title or keyword=TCM OR title or keyword=TCM) OR title or keyword=TCM) OR title or keyword=TCM) OR title or keyword= Chinese patent medicine) AND title or keyword=diabetes))) AND (title or keyword=treatment OR title or keyword=diagnosis and treatment)))) AND ((title or keyword=systematic review OR title or keyword=meta-analysis ) OR title or keyword=meta-analysis)))) |

# Supplementary table 3: Search strategy of Wanfang (234)

| STEPS | Search Terms (in Chinese) |
| --- | --- |
| 1 | Title or keyword: (Chinese medicine or Chinese herbal medicine or Chinese medicine or traditional Chinese medicine or Chinese patent medicine) and title or keyword: (diabetes) and title or keyword: (treatment or diagnosis and treatment) and title or keyword: (systematic review or meta-analysis or meta-analysis) |

# Supplementary table 4: Search strategy of PubMed (25)

| STEPS | | Search Terms |
| --- | --- | --- |
| 1 | ((((((Diabetes Mellitus[Title/Abstract]) OR (Diabetes Insipidus[Title/Abstract])) OR (Diet, Diabetic[Title/Abstract])) OR (Prediabetic State[Title/Abstract])) OR (Scleredema Adultorum[Title/Abstract])) OR (Glycation End Products, Advanced[Title/Abstract])) OR (Glucose Intolerance[Title/Abstract]) | |
| 2 | (((((((Medicine, Chinese Traditional[Title/Abstract]) OR (Traditional Chinese Medicine[Title/Abstract])) OR (Chung I Hsueh[Title/Abstract])) OR (Hsueh, Chung I[Title/Abstract])) OR (Traditional Medicine, Chinese[Title/Abstract])) OR (Zhong Yi Xue[Title/Abstract])) OR (Chinese Traditional Medicine[Title/Abstract])) OR (Chinese Medicine, Traditional[Title/Abstract]) | |
| 3 | (((((Therapeutics[Title/Abstract]) OR (Therapeutic[Title/Abstract])) OR (Therapy[Title/Abstract])) OR (Therapies[Title/Abstract])) OR (Treatment[Title/Abstract])) OR (Treatments[Title/Abstract]) | |
| 4 | ((((((Drugs, Chinese Herbal[Title/Abstract]) OR (Chinese Drugs, Plant[Title/Abstract])) OR (Chinese Herbal Drugs[Title/Abstract])) OR (Herbal Drugs, Chinese[Title/Abstract])) OR (Plant Extracts, Chinese[Title/Abstract])) OR (Chinese Plant Extracts[Title/Abstract])) OR (Extracts, Chinese Plant[Title/Abstract]) | |
| 5 | ((((((((Review, Systematic[Title/Abstract]) OR (Systematic Review[Title/Abstract])) OR (Meta Analysis[Title/Abstract])) OR (Data Pooling[Title/Abstract])) OR (Data Poolings[Title/Abstract])) OR (Overviews, Clinical Trial[Title/Abstract])) OR (Clinical Trial Overviews[Title/Abstract])) OR (Clinical Trial Overview[Title/Abstract])) OR (Overview, Clinical Trial[Title/Abstract]) | |
| 6 | #4 or #2 | |
| 7 | #1 AND #3 AND #5 AND #6 | |

# Supplementary table 5: Search strategy of Web of Science (157)

| STEPS | | Search Terms |
| --- | --- | --- |
| 1 | (((TS=(Diabetes Mellitus OR Diabetes Insipidus OR Diet, Diabetic OR Prediabetic State OR Scleredema autorum OR Glycation End Products, Advanced OR Glucose Intolerance)) AND TS=(Medicine, Chinese Traditional OR Traditional Chinese Medicine OR Chung I hsieh OR hsieh, Chung I OR Traditional Medicine, Chinese OR Zhong Yi Xue OR Chinese Traditional Medicine OR Chinese Medicine, Traditional OR Drugs, Chinese Herbal OR Chinese Drugs, Plant OR Chinese Herbal Drugs OR Herbal Drugs, Chinese OR Plant Extracts, Chinese OR Chinese Plant Extracts OR Extracts, Chinese Plant)) AND TS=(Therapeutics OR Therapeutic OR Therapy OR Therapies OR Treatment OR Treatments)) AND TS=(Review, Systematic OR Systematic Review OR Meta Analysis OR Data Pooling OR Data pollings OR Overviews, Clinical Trial OR Clinical Trial Overviews OR Clinical Trial Overview OR Overview, Clinical Trial) | |

# Supplementary table 6: Search strategy of Embase (78)

| STEPS | Search Terms |
| --- | --- |
| 1 | ((Diabetes Mellitus or Diabetes Insipidus or Diet, Diabetic or Prediabetic State or Scleredema Adultorum or Glycation End Products, Advanced or Glucose Intolerance) and (Therapeutics or Therapeutic or Therapy or Therapies or Treatment or Treatments) and (Drugs, Chinese Herbal or Chinese Drugs, Plant or Chinese Herbal Drugs or Herbal Drugs, Chinese or Plant Extracts, Chinese or Chinese Plant Extracts or Extracts, Chinese Plant or Medicine, Chinese Traditional or Traditional Chinese Medicine or Chung I Hsueh or Hsueh, Chung I or Traditional Medicine, Chinese or Zhong Yi Xue or Chinese Traditional Medicine or Chinese Medicine, Traditional) and (Therapeutics orTherapeutic or Therapy or Therapies or Treatment or Treatments) and (Review, Systematic or Systematic Review or Meta Analysis or Data Pooling or Data Poolings or Overviews, Clinical Trial or Clinical Trial Overviews or Clinical Trial Overview or Overview, Clinical Trial)).mp. [mp=title, abstract, heading word, drug trade name, original title, device manufacturer, drug manufacturer, device trade name, keyword heading word, floating subheading word, candidate term word] |

# Supplementary table 7: AMSTAR-2 Scoring Results

| **Author** | Term1 | Term2 | Term3 | Term4 | Term5 | Term6 | Term7 | Term8 | Term9 | Term10 | Term11 | Term12 | Term13 | Term14 | Term15 | Term16 | **Results** |
| --- | --- | --- | --- | --- | --- | --- | --- | --- | --- | --- | --- | --- | --- | --- | --- | --- | --- |
| Zhipeng Hu 2021.[11] | 1 | 1 | 1 | 1 | 1 | 1 | 1 | 1 | 1 | 1 | 1 | 1 | 1 | 1 | 1 | 1 | HIGH |
| Dingyuan Zhong 2016[12] | 1 | 0 | 1 | 1 | 1 | 1 | 0 | 1 | 1 | 0 | 1 | 1 | 1 | 0 | 1 | 0 | VERY LOW |
| Jiarong Lan 2015[13] | 1 | 0 | 1 | 0.5 | 1 | 1 | 0 | 1 | 1 | 0 | 1 | 1 | 1 | 1 | 1 | 1 | VERY LOW |
| Xue Wang 2015[14] | 1 | 0 | 1 | 0.5 | 0 | 0 | 0 | 1 | 1 | 0 | 1 | 0 | 0 | 1 | 1 | 0 | VERY LOW |
| Haibo Huang 2015[15] | 1 | 0 | 1 | 0.5 | 0 | 0 | 0 | 1 | 0 | 0 | 1 | 0 | 0 | 1 | 1 | 0.5 | VERY LOW |
| Yongzhong Wang 2015[16] | 1 | 0 | 1 | 0.5 | 1 | 1 | 0 | 1 | 1 | 0 | 1 | 0 | 0 | 0 | 1 | 0.5 | VERY LOW |
| Xiaodong Han 2014[17] | 1 | 0 | 1 | 1 | 1 | 1 | 1 | 1 | 1 | 0 | 1 | 0 | 0 | 1 | 1 | 0 | VERY LOW |
| Chi Xiao 2014[18] | 1 | 0 | 1 | 1 | 1 | 1 | 0 | 1 | 1 | 0 | 1 | 1 | 1 | 1 | 1 | 0 | VERY LOW |
| Xiu-feng Yan 2014[19] | 1 | 0 | 1 | 1 | 1 | 1 | 0 | 1 | 1 | 0 | 1 | 0 | 0 | 0 | 1 | 0 | VERY LOW |
| Lu Sun 2012[20] | 1 | 1 | 1 | 0 | 1 | 0 | 0 | 1 | 1 | 0 | 1 | 1 | 1 | 1 | 1 | 0 | VERY LOW |
| Winnie Chen 2012[21] | 1 | 1 | 1 | 1 | 1 | 1 | 0 | 1 | 1 | 0 | 1 | 1 | 1 | 1 | 1 | 1 | LOW |
| Jinlan Peng 2013[22] | 1 | 1 | 1 | 1 | 1 | 1 | 0 | 1 | 1 | 0 | 1 | 1 | 1 | 1 | 1 | 0 | LOW |
| Pu Run 2013[23] | 1 | 1 | 1 | 1 | 1 | 1 | 1 | 1 | 1 | 0 | 1 | 1 | 1 | 1 | 1 | 1 | HIGH |
| Luyao Zhang 2019[24] | 1 | 0 | 1 | 0.5 | 1 | 1 | 0.5 | 1 | 1 | 0 | 1 | 1 | 1 | 1 | 0 | 0 | VERY LOW |
| Yongzhong Wang 2014[25] | 1 | 1 | 1 | 1 | 1 | 0 | 0 | 1 | 1 | 0 | 1 | 1 | 1 | 1 | 1 | 0 | LOW |
| Jie Zhu 2018[26] | 1 | 1 | 1 | 1 | 1 | 1 | 1 | 1 | 1 | 0 | 1 | 1 | 1 | 1 | 1 | 1 | HIGH |
| Jingyan Yan 2018[27] | 1 | 0 | 1 | 0.5 | 0 | 0 | 1 | 1 | 1 | 0 | 1 | 1 | 1 | 1 | 1 | 1 | VERY LOW |
| Yuqing Liang 2018[28] | 1 | 0 | 1 | 1 | 1 | 1 | 0.5 | 1 | 1 | 0 | 1 | 1 | 1 | 1 | 1 | 1 | VERY LOW |
| Wei Zhang 2018[29] | 1 | 0 | 1 | 0.5 | 1 | 1 | 0.5 | 1 | 1 | 0 | 1 | 1 | 1 | 1 | 1 | 0 | VERY LOW |
| Yue Cheng 2018[30] | 1 | 0 | 1 | 0.5 | 1 | 1 | 0.5 | 1 | 0 | 0 | 1 | 0 | 1 | 1 | 1 | 0 | VERY LOW |
| Chongqi Ma 2017[31] | 1 | 0 | 0 | 0 | 0 | 0 | 0 | 1 | 1 | 0 | 1 | 1 | 0 | 0 | 1 | 0 | VERY LOW |
| Linlin Kong 2012[78] | 1 | 0 | 0 | 1 | 1 | 1 | 0 | 1 | 1 | 1 | 0 | 0 | 0 | 1 | 0 | 1 | VERY LOW |
| Tingting Shao 2009[33] | 1 | 0 | 0 | 1 | 1 | 1 | 0 | 1 | 1 | 1 | 0 | 0 | 0 | 1 | 0 | 1 | VERY LOW |
| Dugoua Jean-Jacques 2007[34] | 1 | 0 | 1 | 0.5 | 1 | 1 | 0 | 1 | 1 | 0 | 1 | 1 | 1 | 1 | 1 | 1 | VERY LOW |
| X. Wang 2014[35] | 1 | 0 | 1 | 1 | 1 | 1 | 0.5 | 1 | 1 | 0 | 1 | 0 | 1 | 0 | 0 | 1 | VERY LOW |
| Xiaolin Zhang 2020[36] | 1 | 1 | 1 | 1 | 1 | 1 | 0 | 1 | 1 | 1 | 1 | 1 | 1 | 1 | 1 | 1 | LOW |
| Chunli Piao 2020[37] | 1 | 1 | 1 | 1 | 1 | 1 | 0 | 1 | 1 | 0 | 1 | 1 | 1 | 0 | 1 | 0 | LOW |
| Xitao Ma [38] | 1 | 0 | 1 | 1 | 0 | 0 | 0 | 1 | 1 | 0 | 1 | 1 | 1 | 1 | 0 | 0 | VERY LOW |
| Jiahui Hu 2020[39] | 1 | 0 | 1 | 1 | 1 | 1 | 0 | 1 | 1 | 1 | 1 | 1 | 1 | 1 | 1 | 0 | VERY LOW |
| Jiahui Hu 2020[40] | 1 | 0 | 1 | 1 | 2 | 2 | 0 | 1 | 1 | 1 | 1 | 1 | 1 | 1 | 0 | 0 | VERY LOW |
| Ying Fu 2020[41] | 1 | 0 | 1 | 1 | 0 | 0 | 0 | 1 | 0 | 0 | 1 | 1 | 1 | 1 | 1 | 0 | VERY LOW |
| Huijuan Gao 2019[42] | 1 | 1 | 1 | 1 | 1 | 1 | 0 | 1 | 1 | 0 | 1 | 1 | 1 | 1 | 1 | 0 | LOW |
| Fangyan Huang 2019[43] | 1 | 0 | 1 | 1 | 1 | 1 | 0 | 1 | 1 | 0 | 1 | 1 | 1 | 1 | 0 | 0 | VERY LOW |
| Fengmei Lian 2019[44] | 1 | 1 | 1 | 1 | 1 | 1 | 0 | 1 | 1 | 0 | 1 | 1 | 1 | 1 | 1 | 1 | LOW |
| Yujiao Zheng 2021[45] | 1 | 1 | 1 | 0.5 | 1 | 1 | 0 | 1 | 1 | 1 | 0 | 0 | 0 | 1 | 0 | 1 | VERY LOW |
| Xu Zhou 2021[46] | 1 | 1 | 1 | 1 | 1 | 1 | 0 | 1 | 1 | 1 | 1 | 1 | 1 | 1 | 1 | 1 | LOW |
| Zhipeng Hu 2021[47] | 1 | 1 | 1 | 1 | 1 | 1 | 1 | 1 | 1 | 0 | 1 | 1 | 1 | 1 | 1 | 1 | HIGH |
| Jiang Li 2021[48] | 1 | 1 | 1 | 1 | 1 | 1 | 0 | 1 | 1 | 1 | 1 | 1 | 1 | 1 | 1 | 1 | LOW |
| Guohua Mu 2021[49] | 1 | 0 | 0 | 1 | 1 | 1 | 0 | 1 | 1 | 0 | 1 | 0 | 0 | 1 | 0 | 0 | VERY LOW |
| Aiping Deng 2021[50] | 1 | 0 | 1 | 0 | 1 | 1 | 0 | 1 | 1 | 1 | 1 | 1 | 1 | 0 | 1 | 0 | VERY LOW |
| Zhiyuan Deng 2020[51] | 0 | 0 | 1 | 0.5 | 1 | 1 | 0 | 1 | 1 | 1 | 1 | 1 | 1 | 0 | 1 | 0 | VERY LOW |
| Jiaxing Tian 2019[52] | 1 | 0 | 1 | 0.5 | 0 | 0 | 0.5 | 0.5 | 0 | 1 | 1 | 0 | 0 | 1 | 1 | 1 | VERY LOW |
| Siyi Zhao 2019[53] | 1 | 0 | 1 | 1 | 1 | 1 | 0.5 | 1 | 1 | 1 | 1 | 1 | 1 | 1 | 1 | 1 | LOW |
| Tingting Guo[54] | 1 | 0 | 1 | 0.5 | 0 | 0 | 0.5 | 0 | 0 | 0 | 0 | 0 | 0 | 0 | 0 | 0 | VERY LOW |
| Huiping Tian 2019[55] | 1 | 0 | 1 | 0.5 | 0 | 0 | 0.5 | 1 | 1 | 1 | 1 | 1 | 1 | 1 | 1 | 0 | LOW |
| Yanling Dai 2019[56] | 1 | 0 | 1 | 0.5 | 1 | 1 | 0.5 | 1 | 1 | 1 | 1 | 1 | 1 | 1 | 0 | 0 | VERY LOW |
| Yuming Gu 2018[57] | 1 | 1 | 1 | 0.5 | 1 | 1 | 0.5 | 0.5 | 1 | 1 | 0 | 1 | 1 | 1 | 0 | 1 | VERY LOW |
